# Irregular Dentition

**Published:** 1871-01

**Authors:** 


### IRREGULAR DENTITION.

Extract of Clinic from Dr. J. E. GARRETSON, Lecturer on Surgical Diseases of the Mouth.

I next show you a girl, 15 years of age, who comes to us with an irregular, uneven superior dental arch; and as it illustrates some of the anomalies of dentition, I bring the case before you. We look at her upper jaw, and perceive that she has sixteen teeth, eight upon either side of the center. Now she is only 15 years old, at which age you know, that the ten deciduous teeth should have all been replaced by permanent teeth, yet the third molars or wisdom teeth should not have appeared. Her complete set upon the upper jaw, at present, would therefore be fourteen. I find that there are here three molars upon each side, yet there is space still remaining behind these, in the normal position of the wisdom teeth, and am therefore confident that these are still to erupt, which would give her eighteen in the arch. Whence then these three molars? I examine them carefully. The posterior one is evidently the normal second molar, and the next neighbor is the ordinary first molar; yet between this and the bicuspids is an intruder upon either side? What is it?

Supernumerary teeth are, as a rule, doubly conoidal; but this is not so. It is, however, small and diminutive, and presents every appearance of a milk tooth—the second molar; and such in truth it is, remaining here at the fifteenth year, whereas it should have dropped out at the tenth—its fangs not having been absorbed from some reason unknown to us. Now, fortunately, there has been nearly sufficient room in her arch to accommodate this retention, and the teeth are but moderately displaced; yet, suppose that it had been impossible for the permanent teeth to have crowded their way into the arch,—they must have been compelled to make alveoli for themselves, and would, probably, have emerged posteriorly to the deciduous, at some point in the roof of the mouth, or might have formed an odontocele. This condition of odontocele is one we will frequently remark when we find a deciduous tooth in the place of a permanent one, and, in fact, we should bear this relation in mind in all dentigerous cysts, for they are often found in connection also with supernumerary teeth.

In its relation to these osteo-dental, or dentigerous tumors, or odontoceles, or compound cysts, as they are variosly called, this case is especially interesting to us, since the presence of these teeth might have readily resulted in the formation of such a tumor. The most common position for such tumors is in the region of the palatine processes of the superior maxilla. Yet, I remember one case in which I found such an encysted tooth, with its apex in immediate relation with the floor of the orbit. Your diagnosis will rest mainly upon their position and upon their size, but in cases of obscurity you could easily settle the question by an exploring needle, which will reveal, at least to an educated touch, the presence or absence of tooth structure. The contents of these tumors, or compound cysts, sometimes consist of undeveloped but more commonly of supernumerary teeth. The presence of these supernumerary teeth in such a position is interesting also to us in a physiological as well as a surgical point of view, for as they are not necessarily a dermatic production, the appearance of these in the mouth is as unaccountable as is their association with ovarian or other remote tumors.

In the present instance, however, these teeth are nearly in position, as are also the permanent, so that we have but to afford a little more room in the arch, by the extraction of these offending bodies, thus allowing the remaining ones to fall into their normal place.

An encysted tooth germ, as could readily be inferred, may heterogeneously develop; that is, there may be such a transposition of the dental elements that the microscope can alone individualize them. A famous case of this kind I have reported in my Oral Surgery, where the son of a banker, as the result of encystment of the germs of two of the molar teeth, had a tumor form in his lower jaw, which never was diagnosed until after a resection of the bone, when the microscope demonstrated it to be dentigerous. Anomalies of dentition should claim from you considerable attention.

